# Blockchain Technology for Healthcare: Facilitating the Transition to Patient-Driven Interoperability

**DOI:** 10.1016/j.csbj.2018.06.003

**Published:** 2018-06-30

**Authors:** William J. Gordon, Christian Catalini

**Affiliations:** a Brigham and Women's Hospital, 41 Avenue Louis Pasteur #209, Boston, MA 02115, United States; b Massachusetts General Hospital, 55 Fruit St, Boston, MA 02114, United States; c Harvard Medical School, 25 Shattuck St, Boston, MA 02115, United States; d MIT Sloan School of Management, 30 Memorial Drive, Cambridge, MA 02142, United States; e National Bureau of Economic Research, 1050 Massachusetts Ave., Cambridge, MA 02138, United States

**Keywords:** Clinical data, Interoperability, Data exchange, Blockchain, Application programming interface

## Abstract

Interoperability in healthcare has traditionally been focused around data exchange between business entities, for example, different hospital systems. However, there has been a recent push towards patient-driven interoperability, in which health data exchange is patient-mediated and patient-driven. Patient-centered interoperability, however, brings with it new challenges and requirements around security and privacy, technology, incentives, and governance that must be addressed for this type of data sharing to succeed at scale. In this paper, we look at how blockchain technology might facilitate this transition through five mechanisms: (1) digital access rules, (2) data aggregation, (3) data liquidity, (4) patient identity, and (5) data immutability. We then look at barriers to blockchain-enabled patient-driven interoperability, specifically clinical data transaction volume, privacy and security, patient engagement, and incentives. We conclude by noting that while patient-driving interoperability is an exciting trend in healthcare, given these challenges, it remains to be seen whether blockchain can facilitate the transition from institution-centric to patient-centric data sharing.

## 1 Introduction

The 2009 Health Information Technology for Economic and Clinical Health Act (HITECH), part of the American Recovery and Reinvestment Act, earmarked almost $30 billion in funds to incentivize Electronic Health Record (EHR) adoption by US healthcare providers, largely through the “Meaningful Use” (MU) program [1]. As a result of this effort, providers and hospital use of EHRs has increased dramatically—while only 9% of non-federal acute care hospitals had a basic EHR in 2008, 96% had an EHR by 2015 (with basic EHR defined as a set of 10 core measures including clinician notes, medication lists, and problem lists, among others) [2]. Unfortunately, while the digitization of health records has clearly increased, sharing electronic health data between different hospitals and providers has lagged behind EHR adoption, for numerous reasons, including technical, operational, and privacy-related concerns [1, [3], [4], [5]].

Interoperability in healthcare is often focused around data exchange between business entities—for example, multiple hospital systems through a state-wide Health Information Exchange (HIE) [6]. However, there has been a recent push towards patient-driven interoperability, in which health data exchange is patient-mediated and patient-driven. Notable recent efforts in this area include the 21st Century Cures Act's (21CCA) emphasis on Application Programming Interfaces (APIs) [7], the API requirement in MU stage 3, and recent announcements supporting open APIs from the Department of Veterans Affairs (VA) [8] and from the Center for Medicare and Medicaid Services (CMS) [9].

The shift towards patient-centered interoperability is an important trend that has the potential to lay new groundwork for data sharing in healthcare. Patient-centered interoperability, however, brings with it new challenges and requirements around security and privacy, technology, incentives, and governance that must be addressed for this type of data sharing to succeed at scale, and many of these challenges are still not solved for traditional interoperability [10]. Thus, it is appropriate to look for novel or disruptive interventions that could be applicable in facilitating the shift to patient-centered interoperability. Such interventions could ease the tension between the advantages of data liquidity—clinical, research, operational—and the substantial barriers to interoperability that define the landscape of health data sharing.

Blockchain is one such novel technology that could have a role in improving interoperability. Blockchain—described in detail elsewhere [11, 12]—has particular appeal to health data given its emphasis on sharing, distribution, and encryption. In particular, newer blockchain efforts—smart contracts, second-layer systems, permissioned blockchains— further the potential health care use-cases, and there has been no shortage of hype surrounding the potential of the technology within healthcare [13]. In this work, we describe the health data interoperability problem, and the shift from institution-driven interoperability to patient-centered interoperability. We look at potential ways blockchain could facilitate this transition and benefit interoperability in general. Finally, we close by noting the often substantial limitations around these approaches, as well as appropriate next steps.

## 2 Interoperability: Current State

The Health Information and Management Systems Society defines interoperability as “the ability of different information technology systems and software applications to communicate, exchange data, and use the information that has been exchanged” [14]. For healthcare, interoperability has several potential benefits. First, well-communicating systems can improve operational efficiency, reducing time spent on administrative tasks like manually entering data received from faxes [15]. Interoperability can also reduce duplicate clinical interventions like imaging studies or lab orders, decreasing overall health system cost, decreasing waste, and improving patient safety by reducing the exposure to radiation or invasive procedures [16, 17]. Finally, interoperability may also improve clinical care, by facilitating improved access to relevant, longitudinal clinical data at the point-of-care [18]. While there are mixed results from empirical studies looking at specific interoperable implementations, for example, state-level HIEs [6], the overall goal of interoperability is a necessary component of cost-effective, comprehensive clinical care.

**Fig. 1:**
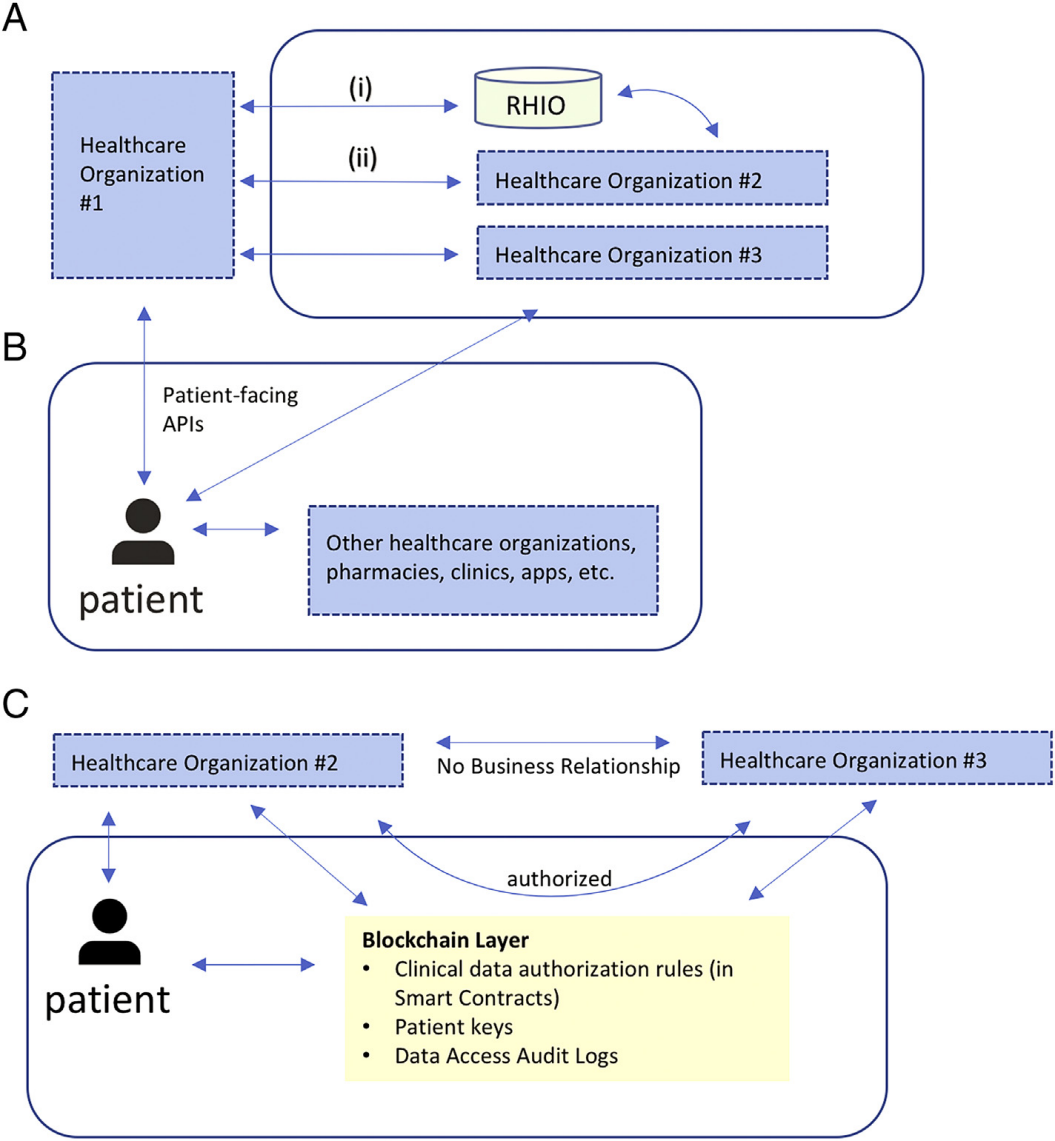
(A) Example of institution-driven interoperability for clinical EHR data. Bi-directional clinical data exchange occurs (i) through an intermediary like a Regional Health Information Organization (RHIO) or (ii) directly between health care organizations with specific business agreements. In both cases, data interfaces are entity-to-entity, not entity-to-patient. In this example, since organization #2 and #3 do not have a specific relationship, there is no bi-directional data flow; providers from organization #2 can request data from organization #3 via one-off requests (like a fax). If a patient receives care at all three organizations, their health data will be scattered across all three EHRs. (B) Example of patient-driven interoperability. Data sharing centers on the patient; in this example, using patient-facing APIs, a patient can directly retrieve their clinical EHR data from organization #1 and organization #3. Once retrieved, the patient can share with other organizations directly. Data flow can be bidirectional. RHIOs and entity-to-entity relationships may still exist as parallel functions. (C) Blockchain-enabled patient-driven interoperability. In this example, the patient can still retrieve data directly from organization #2; however, through blockchain-enabled smart contracts, the patient can authorize sharing of clinical EHR data between organization #2 and organization #3, which do not have a formal business relationship. The blockchain layer stores these authorization rules, along with patient public keys (to ensure entity resolution), as well as data access audit logs. Each organization will manage linking a patient's public key to their own internal enterprise master patient index system independently, and patients can update the smart contract-driven authorization rules as appropriate (for example, adding a new institution if they are seeing a new provider).

The healthcare interoperability landscape is generally centered around business entities, like hospitals, private clinics, and pharmacies, and data is typically created and siloed within the information system that creates it (for example, a hospital's electronic health record) (Fig. 1A). Exchange is often motivated by financial incentives or regulatory pressure [19], and numerous efforts exist to encourage better health data liquidity. For example, 21CCA places a strong emphasis on data sharing [7], and HITECH laid the groundwork for state-wide health-information exchanges, which have also required significant funding [20]. The result of this structure is that an individual patient's health data is scattered across numerous systems, and no institution has a complete picture. Furthermore, even if the different systems were highly interoperable, there would still be missing data—personal device monitor data, lifestyle behavior, social determinants of health—that is generated by patients. The EHR representation of a patient is often the closest approximation of a complete picture that exists in one place, and there has been recent interest in bringing in additional data to EHRs, in particular the social and behavioral determinants of health, to address this limitation [[21], [22], [23]].

Additionally, there are numerous challenges to interoperability that persist. Exchange between different institutions can be operationally challenging, and requires significant collaboration between the entities involved. Data sharing agreements, complex patient matching algorithms, procedures, and governance rules are just some of the issues that need to be agreed upon before data exchange can take place [24]. There are also numerous technical barriers. For example, transactional and entity authentication must be robust (and repeated for every entity-to-entity relationship.) Activity and threshold monitoring, along with some anomaly detection, should also be in place. Finally, the security of data exchange is paramount, and standards for data exchange (for example, FHIR or CDA [25]) must also be agreed upon.

In this setting, there has been a burst of recent energy towards improving the ability of patients to access their own health data. There is little ambiguity about whether patients should be able to access their health data—HIPAA requires that covered entities provide individuals with access to their health data upon request (with certain exceptions, like psychotherapy notes) [26]. While this has traditionally been handled by organizational Health Information Management offices through photocopies and faxes, electronic data access is now heavily regulated through efforts like Meaningful Use (which requires that has a patient has the ability to view, download, and transmit their health information, as well as access their health information through an API [27]) and 21CCA, which actually legislates an API requirement for EHR system certification [7]. Patient portals continue to provide patients with electronic access to their results and other documentation [28], and taking the API functionality a step further, the CMS and VA recently announced new initiatives to further improve patient access to their electronic health data [8, 9]. Clinical data standards like Fast Healthcare Interoperability Resources (FHIR), as well as practical implementation consortiums like the Argonaut project, will further reduce barriers to data exchange [29].

As data liquidity becomes less of a concern through expanded APIs, and as patients obtain better electronic access to their data, they can increasingly become the digital stewards of their health data. The data may still be largely generated in institutional silos, but, patients will now have the ability to build a comprehensive view of their health, retrieving their data and sharing it as appropriate with other entities (Fig. 1B). The transition to patient-driven interoperability will require new processes around security protocols, privacy configurations, electronic consent, and governance. Next, we look at how blockchain technology could intervene and provide benefit in this transition.

## 3 Reducing the Cost of Verification and Networking

The key features of blockchain technology are described in detail elsewhere [[11], [12], [13]]. In brief, blockchain technology can allow multiple stakeholders to agree, at regular intervals, about the true state of shared data. Such shared data can represent credentials and attributes of transactions, information about individuals, entities etc. Depending on how the technology is designed and implemented, it can also take advantage of incentives to drive contributions, manage updates and reconcile records—for example, offering a monetary reward for network participation, thus further incentivizing user engagement. “Smart Contracts” are an important component of platforms such as Ethereum [30], and enable agreements between parties to be governed and enforced by computer code, which might be stored on a blockchain. Similarly, blockchain can offer different degrees of privacy and anonymity, transparency and immutability of the records. For example, while “bitcoin” is public, a “permissioned” blockchain network might have tighter access controls around consensus mechanisms or smart contract creation by restricting membership and read and write controls. While the most well-known blockchain implementation is the digital currency bitcoin, potential use-cases go well beyond finance, and have become particularly salient for healthcare [13, [31], [32], [33]].

Previous work has used economic theory to describe how blockchain technology will shape innovation, specifically around lowering the *cost of verification* of digital attributes and the *cost of networking* [11]. The first cost refers to blockchain's ability to verify the attributes of a transaction (e.g. did it take place or not, who is involved, what are the credentials of the individuals involved etc.) and ensure data integrity at a lower cost than traditional systems. The second cost refers to the ability to bootstrap and operate a marketplace without relying on traditional intermediaries (like financial institutions, or in the case of healthcare data, a Hospital Information Management office). This reduction in market power has a positive effect on competition between different entities that operate within the same market, can allow for a greater degree of data privacy, and lowers barriers to entry for new players. Both costs play a key role in understanding how patient-driven interoperability could be strengthened through blockchain technology.

All clinical data transactions have verification costs associated with them. There is the cost of securing the data and following regulatory guidelines like HIPAA, along with the actual cost of maintaining a primary source of truth. There are the costs of authenticating different entities and transactions, and there is also the cost of patient matching (and the implicit cost of failing to match, measured in clinical errors, manual effort, and financial cost [34]). Interoperability efforts absorb these costs through various mechanisms—security and privacy personnel, technical support, health information management offices, etc. Similarly, there are important networking costs associated with interoperability—governance and institutional agreements for data sharing, along with similar security and privacy costs associated with joining and operating a network.

Both cost reductions—verification and networking—are important because they can increase the ability of different entities to interoperate and the likelihood and impact of clinical data exchange. A less competitive marketplace would reduce the number of entities sharing data, resulting in less comprehensive clinical data exchange. Similarly, high verification costs might result in adverse clinical events, like missed laboratory results due to improper patient matching [35]. Blockchain could provide an important catalyst for improving data exchange, particularly for patient-driven interoperability.

## 4 Blockchain's Role in Patient-Driven Interoperability

At a high level, blockchain technology can be thought of as a platform for digital exchange, where the platform functions without a traditional intermediary. Health data can live in multiple systems and sharing data requires numerous points of collaboration between entities. As interoperability becomes more patient-centric, there is an opportunity to leverage blockchain technology to facilitate this exchange and give patients greater control over their data. Table 1 highlights these benefits along with healthcare-specific examples.

**Table 1:** Blockchain features that could enable patient-driven interoperability, with examples.

Blockchain Feature	Example of Application to Patient-Driven Interoperability
Digital access rules	Clinical data—stored off-chain or on-chain—is linked to the public key of a patient. The patient can use properties of the blockchain, like smart contracts, to assign access rules for the data. For example, authorizing release to a research patient registry for a fixed period of time.
Data aggregation	A patient connects to different institutional interfaces with institution-specific logins (like a patient portal), and provides that institution with their blockchain public key, along with permission to securely transmit data (or metadata) to the blockchain. Done across multiple institutions, clinical data (or references towards clinical data) can thus be aggregated using the technology.
Data liquidity	Highly time sensitive clinical data—for example, advanced care planning “code status” or medication allergies, can be published on a public blockchain, ensuring ready, liquid access to this information as appropriate.
Patient identity	Patients can manage their public keys—perhaps through a multi-sig wallet or mobile device—and use the public-key infrastructure (PKI) to establish their identity for retrieving clinical data from the blockchain, as well as adding new information (like home monitoring devices). PKI ensures providers and institutions can trust that the patient is generating the data.
Data immutability	Clinical data (or metadata) is securely distributed across multiple entities, ensuring integrity, lowering the risk of loss, and offering an audit trail (in case of malicious actor). Append-only model of blockchain ensures all providers with access to information have complete clinical picture.

The first way blockchain technology could improve patient-driven interoperability is through management of digital access rules. Appropriating permissions for release of clinical data is a challenging function that is typically controlled by the data silo owner. Blockchains enable a centralized and shared mechanism for the management of authentication and authorization rules surrounding data. For example, a blockchain may have “Smart Properties”—an entity whose ownership is managed through a blockchain—to allow some form of digital property to have clean ownership. The custodian of the data (for example, the patient), is clearly represented on the blockchain, and can subsequently assign access rules and permissions around their data, enabling easier sharing.

A second way blockchain technology could foster patient-driven interoperability is through data availability. As patients move to take more ownership of their health data, one of their first tasks will be to gather all of their clinical data together, for example, by establishing an API connection to every system that has data they would like to use. Once a patient has established these connections, they can then collect and aggregate their health data as appropriate. Such a task might be cumbersome if the patient had to manage this on their own. Yet a blockchain platform could facilitate this—particularly if done in conjunction with blockchain-enabled digital access rules. For example, clinical encounters could be securely broadcast to the network and linked to a patient's anonymous digital identity. If all clinical encounters from all institutions followed this paradigm, a patient would only need to interact with one platform as all their health data would be available through the same protocol and standard. Additionally, patients could publish their own Patient-Generated Health data (PGHD) to a blockchain network. Such PGHD could provide activity monitoring or other personal health data captured outside of formal healthcare setting, provided a patient authorizes release [36, 37].

Rapid access to clinical information is a third major way blockchain technology could improve interoperability in the patient context. For data (or permissions) stored “on chain” this is immediately clear—the immutable ledger makes data permissions clear, and once this is established, parties can exchange data. If the data is not stored on the blockchain, but there is meta-data about the primary data—for example, timing, or location—then this also enables streamlined access. Knowing that a patient had an imaging study at a specific hospital in a specific year is helpful, as inquiry can be focused on that resource, even if the imaging study itself is not available. In these ways, blockchain improves data liquidity and data availability, and makes it easier for patients to share their data with other entities.

A fourth way blockchain might facilitate the transition to patient-driven interoperability is around patient identity. There is no US national patient identifier, and clinical information systems frequently have multiple records for one individual. Entity resolution of these records is an active area of operational and research interest, and can be quite challenging at scale [38]. For example, if two different clinical systems interchange clinical data, each must first resolve the patient to an identifier used internally. Blockchain's use of public-key infrastructure (PKI) provides a centralized identification method—an individual's public key—that can be used to link that patient's records across institutions. If each institution knew a patient's public key and the key was linked to their own internal identifier (for example, the patient linked their public key to their patient-portal account when they registered), then subsequent clinical events broadcast to the blockchain network could include that public key as a patient reference, facilitating patient matching.

Finally, it is worth mentioning the importance of immutability. One advantage of traditional, institution-driven interoperability is that patients are not responsible for securing or storing their data—that is left to the entity generating the data. Moving patients to the center of interoperability—even if they are authorizing release on behalf of the entities—has the potential to shift actual data governance away from institutions, particularly for interfaced data not primarily generated by the entity (for example, an HIE). Because blockchains are typically immutable, data added to the chain will persist. This lowers the risk of loss, offers an audit trail (for example, in case of a malicious actor), and ensures that a complete digital history is available to all parties (provided appropriate access controls are in place.)

Fig. 1C describes one approach where two organization without a formal business relationship (but with standard data interfaces) can leverage the blockchain layer for data access and authorization rules. In this example, the blockchain layer also stores data access logs, enabling immutable auditing. Additionally, while this example does not describe the storage of actual clinical data “on chain,” other implementations might put certain clinical data on the blockchain. Such examples could be contextual clinical data (for example, indicating that specific clinical data exists with a pointer towards its location without revealing any content), or even frank encounter data, like a hospitalization event, or specific labs, medications, allergies, problem lists, etc.

## 5 Tensions and Barriers to Blockchain-Enabled Patient-Driven Interoperability

Despite the numerous ways in which blockchain might enable patient-led interoperability, there are several barriers that will need attention. The first barrier is related to the sheer size and volume of clinical data. High-volume, high-frequency transactions are a cornerstone of clinical data, and the size of clinical data is increasing exponentially with modern advancements in technology [39]. For example, a single cardiac Magnetic Resonance Image can require 200 megabytes in storage [40]. Given the distributed nature of a blockchain, it is not feasible to store this data on-chain with current technology. Additionally, verification of new transactions can take time on blockchains based on proof of work (such as the Bitcoin). These limitations favor low-size, relatively infrequent transactions. There are ways to address this—for example bitcoin's Lightning Network [41], blockchains based on alternative approaches to consensus (e.g. proof of stake) or permissioned blockchains—but further work is needed to understand whether these solutions provide reprieve from the scaling challenges.

A second challenge relates to privacy and security. Some implementations of blockchain technology are pseudonymous—identity is typically obscured behind a public key, but other attributes of transactions are publicly shared. This is problematic for health data. First, basic demographic information can identify people [42], and if an individual's public key is matched to their identity, all transactions associated with that public key are then known to be linked to an individual. While catastrophic on a public blockchain, this is also problematic on a private blockchain as an individual may not want all of the members of the private blockchain to have access to the same data, or they may want to revoke authorization to their data at a later point in time, both of which are not possible once their identity is linked to their public key. Blockchain implementations that allow for selective disclosure of private information (e.g. such as Zcash) and rely on zero knowledge cryptography to provide verification of transactions with a high degree of privacy over the underlying data will be needed within the healthcare industry. Importantly, the European Union's (EU) recently adopted General Data Protection Regulation includes a “right to erasure” and places health data in a special category of personal data. This further raises additional questions about the intersection of blockchain, encrypted data, and patient privacy, particularly in the EU context [43].

Related to but separate from privacy and security is the challenge of patient engagement. A patient-driven interoperability framework necessarily involves more patient participation than an institution-driven construct. If a patient receives care at one hospital, and they seek outside records from another institution, a patient may simply sign a form and their providers will facilitate the exchange (electronic or otherwise). If patients are to become autonomous digital stewards, they will need some way of managing their digital assets, for example, a key, or a password. Mechanisms to manage lost digital assets—forgotten passwords, lost keys, etc., will need thought. This could require additional intermediaries, and it is not immediately clear who would play this role—perhaps an opportunity for a new commercial market, akin to cryptocurrency exchanges.

Possibly the largest barrier to adoption relates to incentives. Though EHRs are now required by law to have patient-facing APIs, the same is not true for all healthcare data, and incentivizing institutions to build patient-facing data connections without financial motivation to do so will be challenging—the difference between compliance and true interoperability. For example, while a medication list may be a required data output of a hospital's patient-facing EHR API, it is not clear that a pharmacy benefits manager needs to structure and expose every medication approval or transaction without clear business value. Further incentives around data sharing will further strengthen the API economy and lead to more patient-data autonomy. Table 2 describes these challenges along with potential mitigations.

**Table 2:** Barriers to blockchain-enabled patient-driven interoperability with mitigation.

Challenge	Mitigation
Transaction volume of clinical data	• Focus data exchange on summarized clinical data; for example, a radiology report instead of full DICOM images • Permissioned blockchains for local geographies built to handle large transaction volumes without time-intensive validation • New technologies and research in blockchain scaling methodologies
Privacy and security	• Permissioned, member-only blockchain consortium to minimize public exposure • Data storage off-chain, with on-chain focused around permissions or other meta-data
Patient engagement	• Patient-friendly “app” ecosystem of intermediaries to manage public keys and permissions
Incentives	• Continued federal incentives to expand API coverage, like VA data pledge • Association of open data with value for reimbursement • Competitive pressure of API-enabled systems to encourage non-enabled systems to invest in API infrastructure

## 6 Related Work

Blockchain's potential to enable better health data sharing and ownership has been previously described by several authors. Using a public or private blockchain to actually store clinical data is one example—for example, Yue et al. described a “Healthcare Data Gateway” (HDG) which would enable patients to manage their own health data stored on a private blockchain [44]. Similarly, Ivan described a public blockchain implementation, where healthcare data is encrypted but stored publicly, creating a blockchain-based Personal Health Record [45]. MedChain is another example, where a permissioned network of medication stakeholders (including the patient) could be used to facilitate medication-specific data sharing between patients, hospitals, and pharmacies [46]. While we imagine that a model storing actual clinical data on a blockchain—permissioned or public—would have substantial privacy and scalability concerns, it is important to continue to understand the privacy and security implications of on-chain data storage.

Another approach to sharing health data leverages blockchain not for the storage of the actual clinical data, but for facilitating management or governance of that data. Zyskind et al. have described a general-purpose decentralized access and control manager for encrypted off-chain data; the blockchain layer enforces access control policies, but data is stored off chain [47]. In the healthcare space, FHIRChain is a smart-contract based system for exchanging health data based on the standard FHIR [48], where clinical data is stored off chain, and the blockchain itself stores encrypted meta-data which serve as pointers to the primary data source (like an EHR) [49]. Azaria et al. introduced MedRec, which uses a permissioned blockchain network to facilitate data sharing and authentication. MedRec has a novel proof-of-work incentive method built around access to anonymized medical data (for research, as an example) [50]. Finally, Dubovitskaya et al. also propose a permissioned blockchain (focused on oncologic care) which leverages off-chain cloud storage for clinical data, using the blockchain to manage consent and authorization [51]. Both MedRec and Dubovitskaya's work have been prototyped but do not appear to be operational.

Additionally, it is worth noting that in the drive towards patient-driven interoperability, blockchain may not be the only solution. Private, vendor-based solutions may also take hold. For example, Apple recently announced a product that would allow patients to pull their clinical EHR data from participating institutions using APIs (based on FHIR and the Argonaut project specification) [52]. Similarly, Sync 4 Science is a pilot effort to allow patients to contribute their EHR data to research efforts, also through standard APIs, using an authorization workflow (i.e., the data need never be stored or managed by the patient individually) [53]. Though the idea of a digital Personal Health Record has been described for decades, there has been noticeable traction from a technology and regulatory perspective in recent years.

## 7 Summary and Outlook

This paper describes two types of healthcare interoperability: institution-driven and patient-driven. Institution-driven interoperability, which has historically been the main focus of interoperability efforts, relies on different healthcare entities exchanging data based on business or regulatory incentives. There has been an increasing move towards patient-driven interoperability, in which an individual patient's electronic health data is made available to them through standard mechanisms like APIs. Regulatory pressure in the form of provider incentives like Meaningful Use, heavy financial penalties and required data sharing from 21CCA [7], and patient-focused open-data efforts from CMS and the VA are aligning to create an environment where patients have on-demand access to their health data. In this model, patients are the digital stewards of their health data, authorizing release and sharing to trusted entities.

The shift towards patient-centered interoperability brings with it numerous challenges around patient consent, governance, security, privacy, and patient engagement. Blockchain technology, by creating a platform for the secure exchange of data, is an attractive method of addressing these challenges. We have discussed some of the ways blockchain could facilitate this transition, like digital access rules management, data aggregation, data availability and liquidity, patient identity, and immutability. In essence, blockchain provides a high-level framework for how a patient could securely interact with multiple stakeholders, identify themselves across each entity, and aggregate their health data in a persistent form.

There are practical limitations and challenges around blockchain that will need to be addressed as these areas are explored. Scaling blockchain to support clinical transaction volume is a technical challenge that has garnered significant interest in the blockchain community (since this also affects non-healthcare areas as well). Privacy and security considerations, for example, the anonymous-but-not-private aspect of most blockchain implementations, as well as issues around patient key management and patient engagement, will also need consideration. Finally, incentivizing interoperability will continue to be an issue to ensure continued development and maintenance of patient-facing data interfaces. Future work will need to address these areas. In particular, understanding how to design a patient-centric model that is both usable and useful, while still aligning incentives with multiple stakeholders, will be key. Additionally, as the GDPR is implemented throughout the EU, and privacy laws are updated in other locales, managing data authorization and storage rules will need better clarity.

The shift from institution-driven interoperability to patient-driven interoperability is an exciting trend in healthcare and has the potential to fundamentally alter attitudes and policies around clinical data exchange and ownership. While blockchain technology might have a role in promoting this change, there are numerous challenges that must be addressed before we will see practical implementations. Whether these are surmountable is yet to be seen. Meanwhile, continuing to incentivize patient-facing data exchange will enable patients and providers to shift from an institution-centric to patient-centric data perspective, an important first step in accelerating patient-driven interoperability.

## Declarations of interest

None.
